# Prevention of snus use: Attitudes and activities in the Public Dental Service in the south‐eastern part of Norway

**DOI:** 10.1002/cre2.171

**Published:** 2019-03-13

**Authors:** Tarja Tanner, Håkon Rukke Valen, Liv Grøtvedt, Simen E. Kopperud, Rune Becher, Line Schrøder Karlsen, Vibeke Ansteinsson

**Affiliations:** ^1^ Department of Cariology, Endodontology and Pediatric Dentistry, Research Unit of Oral Health Sciences University of Oulu Oulu Finland; ^2^ Nordic Institute of Dental Materials (NIOM) Oslo Norway; ^3^ Department of Air Pollution and Noise Norwegian Institute of Public Health (NIPH) Oslo Norway; ^4^ Oral Health Centre of Expertise in Eastern Norway (OHCE) Oslo Norway

**Keywords:** health promotion, Norway, oral health, Public Dental Service, snus cessation

## Abstract

Dental health care professionals have the opportunity to play a key role in tobacco prevention and cessation among adolescents. Snus use has increased in Norway, especially in the age group 16–24, whereas there has been a decline in smoking. This study investigated attitudes and activities related to snus prevention among dental health care professionals working in the Public Dental Service (PDS) in south‐eastern Norway. A web‐based survey with a total of 557 dentists and dental hygienists in seven counties in Norway, with a response rate of 53.5%, was carried out in 2017. Dentists' and dental hygienists' activities regarding preventive snus use intervention were analysed using the chi‐square test. Intervention was measured with a score (1–5) based on four questions. Bivariate and multivariate linear regression analyses were used to investigate the associations between the explanatory variables of attitudes/activities and the outcome intervention variable. Approximately 87% of the dentists and 58% of the dental hygienists were not familiar with the “minimum intervention method” for tobacco prevention and cessation. Dental hygienists were most active in informing and supporting their patients in prevention and cessation of snus use. The PDS is an underutilized arena for tobacco prevention and cessation among adolescents, and the intervention potential is particularly high among the dentists.

## 1 INTRODUCTION

The type of smokeless tobacco predominantly used in the Nordic countries is snus, and it is mostly produced in Sweden (Benowitz, 2014; Foulds, Ramstrom, Burke, & Fagerstrom, 2003). The sale of snus is allowed in the European countries of Norway, Belarus, and Russia, but it is illegal everywhere in the European Union except Sweden. Although it most likely has less adverse health effects than smoking, snus use still involves a range of adverse health effects that are not necessarily perceived (by the users). Snus is highly addictive due to its nicotine content; in addition, it contains genotoxic and carcinogenic tobacco‐specific nitrosamines. The use of snus during pregnancy increases the risk of low birth weight, premature birth, and stillbirth (Wikstrom, Cnattingius, Galanti, Kieler, & Stephansson, 2010). Current data support that snus use increases the risk of diabetes type 2 in a dose‐dependent way (Baba, Wikstrom, Stephansson, & Cnattingius, 2012; Baba, Wikstrom, Stephansson, & Cnattingius, 2014). Discontinuing use of snus after a heart attack or stroke of any cause has been shown to reduce mortality by approximately 50% (Arefalk et al., 2014). In addition, there is lack of knowledge regarding adverse health impacts associated with early initiation of snus use. For dentist and dental hygienists, it is noteworthy that snus also affects the oral mucosa, usually causing red or white areas, and wrinkled surfaces called hyperkeratotic lesions. Most often, these snus‐induced lesions heal after cessation of snus use. However, snus may cause permanent retraction of gingiva at the areas where pinches of snus are placed (Roosaar, Johansson, Sandborgh‐Englund, Nyren, & Axell, 2006).

In Norway, snus consumption has increased over the last 10–20 years, especially among young adults. In 2017, 32% of men and 22% of women aged 16–24 used snus on a daily or occasional basis. Concurrently smoking has decreased in the same age group, 3% smoked daily and 14% occasionally, with smaller gender differences than for snus use (Statistics Norway, 2018; Pedersen & von Soest, 2014). A similar increase in snus use among young adults, especially among men, has also been observed in Finland (Kinnunen et al., 2017; Tanner et al., 2014). Among 18‐year‐old Finnish men, 23% use snus daily or occasionally, whereas the corresponding figure among 18‐year‐old Finnish women is 4% (Kinnunen et al., 2017). The prevalence of daily or occasional snus use in Sweden in the age group 16–29 years is 21% for men and 9% for women, respectively (Public health agency Sweden, 2018). However, use of smokeless tobacco is not just a concern in Scandinavian countries; in the United States, 6.6% of men and 0.5% of women were currently smokeless tobacco users in 2016 (Centre of Disease Control, 2016).

Interventions for tobacco prevention and cessation have been studied worldwide (West et al., 2015). School tobacco intervention programmes that combine social competence and social influences to prevent the onset of smoking have shown considerable effects at 1‐year follow‐up and longer (Josendal, Aaro, Torsheim, & Rasbash, 2005; Thomas, McLellan, & Perera, 2015). In addition, both brief and extensive interventions in dental settings indicate positive outcomes on both smoking and snus cessation (Nohlert, Ohrvik, Tegelberg, Tillgren, & Helgason, 2013; Virtanen, Zeebari, Rohyo, & Galanti, 2015; West et al., 2015). Interestingly, stronger intervention effects were observed among snus users compared with smokers in several of the studies (Carr & Ebbert, 2007; Virtanen et al., 2015). One brief method used by health professionals is the minimal intervention method, which is commonly used in tobacco cessation (Towns, DiFranza, Jayasuriya, Marshall, & Shah, 2017). The method consists of three steps: First, the patients are questioned about own tobacco use followed second by question about experience/reflections on own tobacco use and third by recommendation to quit and an offer of cessation support.

In Norway, children and youth up to the age of 18 are entitled to free dental care, whereas patients up to age 20 are offered treatment at a reduced price when visiting the Public Dental Service (PDS). Hence, the PDS could be an ideal arena for tobacco preventive and cessation initiatives among children and youth. According to a study nearly two decades back in time, tobacco intervention activity in Norwegian PDS was not performed on a regular basis (Lund, Lund, & Rise, 2004). Although snus use has increased markedly in several countries, few studies have since focused on snus use prevention activities in dental health care.

The high level of snus use among youth in Norway illustrates the need for preventive measures, as well as tobacco cessation interventions, aimed at this group. The aim of the present study was to investigate public dental health professionals' attitudes and activities regarding interventions towards young patients' snus use in south‐eastern Norway.

## 2 MATERIAL AND METHODS

### 2.1 Study population

Between March and June 2016, a precoded questionnaire was sent electronically to all dentists and dental hygienists in the PDS in seven counties in Norway (Østfold, Hedmark, Oslo, Oppland, Vestfold, Vest‐Agder, and Aust‐Agder). Two reminders were sent to all participants. The participants were asked not to respond more than once. The data were collected using web‐based easy research, a questback product from https://www.questback.com/no/. Participation in the survey was voluntary, and the questionnaire software (easy research) ensured anonymity of the participants. The study population consisted of a gross sample of 388 dentists and 169 dental hygienists. The total response rate was 53.5% (298 participants). Due to missing values, the number of participants varied between 294 and 298 for the variables shown in the tables.

### 2.2 Questionnaire and measures

The questionnaire was composed of 19 questions, based on a questionnaire previously used in studies among dental professionals and general practitioners (Helgason & Lund, 2002; Helgason, Lund, Adolfsson, & Axelsson, 2003; Lund et al., 2004). The wording and answer categories of the questions used in the present study (17 of 19) are shown in Tables 1, 2, 3. Questions 1–4 (Q1–Q4) are about demographic data of dental health personnel, Q5 about the history of own snus use, and Q6 at which age do they start to ask their patients about snus use. Q7–Q10 are included as intervention variables (see Section 2.3), Q11–Q12 about the method of minimal intervention and Q13–Q17 about attitudes and activities in snus use prevention (see Section 2.3).

**Table 1:** Descriptive information about the study population

	Dentists % (*n*)	Dental hygienists % (*n*)	Total % (*n*)
Response rate	52.6 (204)	55.6 (94)	53.4 (298)
Age group (Q1)			
≤25 years	2.5 (5)	8.5 (8)	4.4 (13)
26–45 years	70.6 (144)	50.0 (47)	64.1 (191)
46–65 years	25.0 (51)	41.5 (39)	31.5 (90)
>65 years	2.0 (4)	0	1.3 (4)
Number of years in dental care (Q3)			
10 years or less	61.3 (125)	38.3 (36)	54.0 (161)
11–20 years	19.1 (39)	39.4 (37)	25.5 (76)
20 years or more	19.6 (40)	22.3 (21)	20.5 (61)
History of own snus use (Q5)			
Never	87.7 (179)	86.2 (81)	68.5 (260)
User or former user	12.3 (25)	13.8 (13)	31.5 (38)

**Table 2:** Dentists' and dental hygienists' activities regarding preventive snus use intervention

	Dentists % (*n*)	Dental hygienists % (*n*)	Total % (*n*)	*P* value
At which age do you start to ask about patients' snus habits (Q6)				
10–12 years old	2.0 (4)	2.1 (2)	2.0 (6)	
13–15 years old	23.3 (47)	34.0 (32)	26.7 (79)	
16–18 years old	60.4 (122)	59.6 (56)	60.1 (178)	
>18 years old	14.4 (29)	4.3 (4)	11.1 (33)	<0.020
How often do you ask young patients (younger than 20 years) about their snus habits (Q7)				
Always or often	46.1 (94)	69.1 (65)	53.4 (159)	
Sometimes	38.2 (78)	28.7 (27)	35.2 (105)	
Rarely or never	15.7 (32)	2.1 (2)	11.4 (34)	<0.001
When you treat patients who have snus‐related symptoms in the oral cavity, how often do you ask them about their snus habits? (Q8)				
Always or often	95.1 (194)	94.7 (89)	95.0 (283)	
Sometimes	2.9 (6)	3.2 (3)	3.0 (9)	
Rarely or never	2.0 (4)	2.1 (2)	2.0 (6)	<0.990
When you treat patients who do not have snus‐related symptoms in the oral cavity, how often do you ask them about their snus habits? (Q9)				
Always or often	21.1 (43)	39.4 (37)	26.8 (80)	
Sometimes	24.0 (49)	35.1 (33)	27.5 (82)	
Rarely or never	54.9 (112)	25.5 (24)	45.6 (136)	<0.001
When you learn that a patient uses snus, how often do you record that information in their journal? (Q10)				
Always or often	55.9 (114)	70.2 (66)	60.4 (180)	
Sometimes	23.5 (48)	18.1 (17)	21.8 (65)	
Rarely or never	20.6 (42)	11.7 (11)	17.8 (53)	<0.050
Do you know about the method of Minimal Intervention, which is used in smoking and snus cessation? (Q11)				
Yes	12.7 (26)	42.4 (39)	22.0 (65)	
No	87.3 (178)	57.6 (53)	78.0 (231)	<0.001
Do you use Minimal Intervention to prevent snus use? (Q12)				
Yes	9.8 (20)	40.2 (37)	19.3 (57)	
No	2.9 (6)	2.2 (2)	2.7 (8)	
Missing	87.3 (178)	57.6 (53)	78.0 (231)	<0.050

**Table 3:** Dentists' and dental hygienists' attitudes and activities

	Dentists % (*n*)	Dental hygienists % (*n*)	Total % (*n*)	*P* value
I do not feel I know enough about the effects of snus use on dental health (Q13)				
Agree	39.2 (80)	38.0 (35)	38.9 (115)	
Neutral	20.1 (41)	15.2 (14)	18.6 (55)	
Disagree	40.7 (83)	46.7 (43)	42.6 (126)	0.500
I do not think it is my job to discuss peoples´ snus habits (Q14)				
Agree	6.9 (14)	5.4 (5)	6.4 (19)	
Neutral	8.9 (18)	7.6 (7)	8.5 (25)	
Disagree	84.2 (171)	87.0 (80)	85.1 (251)	0.820
A conversation about snus takes up too much time (Q15)				
Agree	10.8 (22)	7.6 (7)	9.8 (29)	
Neutral	21.2 (43)	9.8 (9)	17.6 (52)	
Disagree	68.0 (138)	82.6 (76)	72.5 (214)	0.020
Snus use is not a major cause of oral health problems (Q16)				
Agree	12.8 (26)	4.3 (4)	10.2 (30)	
Neutral	19.7 (40)	16.3 (15)	18.6 (55)	
Disagree	67.5 (137)	79.3 (73)	71.2 (210)	0.030
I feel awkward about asking people about their snus habits (Q17)				
Agree	18.3 (37)	12.0 (11)	16.3 (48)	
Neutral	12.4 (25)	13.0 (12)	12.6 (37)	
Disagree	69.3 (140)	75.0 (69)	71.1 (209)	0.370

### 2.3 Statistical analysis

For bivariate crosstabs analyses, chi‐square tests were used (*P* value = <0.05). A new intervention variable was constructed with factor analyses from four of the questions in Table 2 (Q7–Q10, with Cronbach's alpha 0.77), given a scale from 1 to 5, where score 1 indicates low and score 5 indicates high intervention. The 5‐point scales for the variables of attitudes/activities (1 = *completely agree* to 5 = *completely disagree*) were collapsed to three categories in Table 3 (Q13–Q17) but treated as continuous scales in the regression analyses in Table 4 (Q13–Q17).

**Table 4:** Linear regression analyses with associations between attitudes/activities and preventive snus use intervention for dentists and dental hygienists

	Dentists (*n* = 204)						Dental hygienists (*n* = 94)					
	Intervention, unadjusted			Intervention, adjusted			Intervention, unadjusted			Intervention, adjusted		
Explanatory variables	*b* [Link]	95% CI	*P* value	*b* [Link]	95% CI	*P* value	*b* [Link]	95% CI	*P* value	*b* [Link]	95% CI	*P* value
I do not feel I know enough about the effects of snus use on dental health (Q13)	−0.072	[−0.146, 0.003]	0.059	−0.018	[−0.091, 0.055]	0.628	−0.149	[−0.251, −0.046]	0.005	−0.082	[−0.198, 0.035]	0.169
I do not think it is my job to discuss peoples´ snus habits (Q14)	−0.230	[−0.325, −0.134]	0.001	−0.163	[−0.267, −0.060]	0.002	−0.242	[−0.386, −0.098]	0.001	−0.205	[−0.374, −0.036]	0.018
A conversation about snus takes up too much time (Q15)	−0.092	[−0.180, −0.004]	0.040	0.011	[−0.078, 0.101]	0.802	−0.058	[−0.199, 0.083]	0.416	0.071	[−0.085, 0.226]	0.367
Snus use is not a major cause of oral health problems (Q16)	−0.150	[−0.236, −0.063]	0.001	−0.089	[−0.176, −0.001]	0.046	−0.049	[−0.206, 0.109]	0.542	−0.028	[−0.179, 0.123]	0.712
I feel awkward about asking people about their snus habits (Q17)	−0.100	[−0.176, −0.023]	0.011	−0.099	[−0.174, −0.024]	0.010	−0.112	[−0.236, 0.012]	0.077	−0.057	[−0.194, 0.080]	0.412

Effect‐measure (beta‐coefficient).

Multivariate linear regression analyses were conducted to find the association between the explanatory variables of attitudes/activities (Q13–Q17) and the outcome intervention variable, measuring the practices regarding preventive snus intervention among dental health personnel (Q7–Q10). The analyses were performed separately for dentists and dental hygienists. The results were expressed as unstandardized beta‐coefficients (*b*) and their 95% confidence intervals.

All analyses were performed using the statistical program SPSS (Statistical Package of Social Sciences; SPSS Inc., Chicago, USA, version 23.0).

## 3 ETHICAL ISSUES

The present study is part of a clinical study investigating oral manifestations of snus use among adolescents in south‐eastern Norway, all approved by both the Norwegian Centre for Research Data (project number: 47365) and the Norwegian Regional Ethical Committee (project number 2015/445).

## 4 RESULTS

A total of 204 dentists (52.6%) and 94 dental hygienists (55.6%) responded to the questionnaire after two reminders, with a total response rate of 53.4%. The dentists were younger than the dental hygienists; 50% of the dental hygienists and 70% of the dentists were between 26 and 45 years of age. More than one tenth of dentists and dental hygienists were snus users or former users (Table 1).

The distributions of intervention practices towards snus use for dentists and dental hygienists are shown in Table 2. Most dentists and dental hygienists started to ask patients about their snus habits at age 16–18 years. Only 2% of dental professionals started to ask their patients about snus use from the age of 10–12 years. From 16 years of age, one fourth of the dentists and more than one third of the dental hygienists started to ask about snus use (Table 2). Nearly all (95%) dentists and dental hygienists asked about their patients' snus use when they found snus‐related symptoms in the oral cavity. When no snus‐related symptoms were observed, 21.1% of dentists and 39.4% of dental hygienists asked patients about their snus habits (Table 2). More dental hygienists (70.2%) than dentists (55.9%) routinely recorded snus use in the patient records. Among the dentists, 12.7% were familiar with the method of minimal intervention in prevention and cessation of snus use. The corresponding figure for dental hygienists was 42.4%.

The distributions of snus use intervention activities for dentists and dental hygienists are shown in Table 3. Less than half of the dentists (41%) and dental hygienists (47%) perceived that they had sufficient knowledge about the effects of snus use on oral health. A majority (85%) of both professionals perceived discussing snus use with patients as part of their job (Table 3). More dentists than dental hygienists found a conversation about snus use too time‐consuming and did not perceive snus use as a major cause of oral health problems (Table 3).

Table 4 shows the associations between attitudes and activities as explanatory variables, and the intervention variable as outcome variable, shown separately for dentists and dental hygienists. In the unadjusted (bivariate) analyses for dentists, all explanatory variables were negatively associated with the intervention factor. The only explanatory nonsignificant variable was the perceived lack of knowledge regarding snus use and dental health. In the corresponding multivariate analyses for dentists, three of the same variables were negatively associated with dentists' intervention. These three were “it is not my job to discuss peoples' snus habits,” “snus use is not a major cause of oral health problems,” and “I feel awkward asking patients about their snus habits.”

Among the dental hygienists, two explanatory variables were negatively associated with the intervention factor in the bivariate analyses: lack of knowledge about the effects of snus use and “it is not my job to discuss peoples' snus habits.” In the multivariate analyses, only the question concerning occupational task remained negatively associated with preventive snus use intervention among the dental hygienists (Table 4). Mean intervention scores for dentists and dental hygienists were 3.71 (*SD* 0.67) and 4.09 (*SD* 0.65), respectively. Dental professionals' working years, own age, and own snus use were not associated with intervention activity (data not shown).

## 5 DISCUSSION

This study investigated attitudes and activities towards snus intervention in adolescent patients among dentists and dental hygienists in the PDS in south‐eastern Norway. The prevalence of smoking among adolescent is low. At the end of high school, smoking prevalence remains low, whereas the prevalence of snus use increases markedly in later years, with current use (daily or weekly) among 18‐year‐old boys and girls at 24% and 18%, respectively (Bakken, 2017). Most of the dentists and dental hygienists participating in the present study began to ask about snus use habits when the patients were 16–18 years old. This is a vulnerable age for initiation of tobacco use (US Department of Health and Human Services, 2012), and it emphasizes the important role of dental health professionals in tobacco prevention. Nearly all adolescents in Norway visit a dentist or an oral hygienist on a regular basis, due to free public dental care for people under age 18 and at reduced price from age 18 until age 20. Tobacco cessation is associated with improved public health in general and improved oral health, in particular (Helgason & Lund, 2002). Nevertheless, tobacco prevention and cessation strategies do not appear to be performed routinely in dental clinics (Lund et al., 2004). Guidelines for systematic tobacco prevention to youth have not yet been implemented in the PDS in Norway. Based on the high prevalence of snus use among adolescents, tobacco prevention guidelines adapted to dental professionals should be required.

More dental hygienists than dentists were engaged in a conversation about snus use and were concerned about snus use as a hazard for oral health. The different attitudes observed between the oral health professionals towards tobacco intervention could be due to differences in occupational assignment. Dentists may be more treatment‐ and task‐focused compared with dental hygienists, who mainly provide preventive care. The differences probably relate mostly to differences in the educational programmes but may also relate to the organization and allocation of resources in the PDS. Improvements in tobacco prevention may be achieved by allocating responsibility for tobacco prevention to both professionals. Given that dentists have a different approach to patients than dental hygienists, both approaches may have their own value, and a short conversation with the dentist could reinforce the message from the hygienist (An et al., 2008).

Our results are in line with previous studies showing that dental clinics and the oral health professionals are an important but underutilized arena for tobacco prevention and cessation (Helgason et al., 2003; Lund et al., 2004). The reason for this is not known; however, guidelines regarding tobacco prevention and cessation strategies have focused on the primary (physician‐based) health services, and the dental health service has been less involved. This could most likely have an impact on the engagement of dental public health personnel.

An interesting finding in the present study was that almost all participants among the dental professionals perceived talking about snus use as part of their job. However, the majority stated that they lacked sufficient knowledge about the impact of snus use on oral health. It is therefore important to provide information to oral health professionals about localized and general adverse health effects associated with snus use, especially when snus is perceived as a safe alternative to smoking. Changes in the oral mucosa appear frequently, which could make the cessation advice coming from dental professionals particularly relevant to snus users (Virtanen et al., 2015). Also, brief and structured counselling in dentistry was found to be associated with reduction of tobacco use, even when a statistical significant association was not found regarding total abstinence (Virtanen et al., 2015). These results should encourage dental health personnel to continue inform about snus use in the clinic setting.

According to the study by Lund et al. (2004), the most common barrier towards intervention among dentists and dental hygienists were the belief that discussing tobacco use (snus and smoking) was outside their field of responsibility. In addition, dentists also reported that discussing tobacco was too time‐consuming, whereas approximately 1 of 5 of the dental hygienists felt awkward asking the patients about their smoking habits. Lund and co‐workers also reported that dental hygienists intervened more often than dentists. This is in line with the findings of the present study, where more dental hygienists than dentists were familiar with the minimal intervention method and where dental hygienists had a higher intervention score for snus use, compared with dentists. One could speculate that one reason for this discrepancy is the last years education or updated courses of dental hygienists, where the high prevalence of snus use among young Norwegians has been emphasized, more so than in the education of dentists. Interestingly, we found a substantially higher percentage of dental personnel that regarded snus use as an oral health problem compared to the study from 2004 by Lund and co‐workers. This could be due to increased focus and knowledge about snus and health in general. Brief intervention methods such as minimal intervention can be effective in reducing the prevalence of tobacco use when used by health professionals in general (Stead et al., 2013). Furthermore, interventions from multiple health professionals are believed to have a positive effect on tobacco prevention and cessation (World Health Organization, 2005). In our opinion, both professionals have an important responsibility in tobacco prevention and cessation and should share the opportunity to do this work, in a complementary manner.

Advantages of the present study included recent data collection (2016), the inclusion of public dental clinics in both urban and rural areas and almost 40% of the employed dentist and dental hygienists in the PDS in Norway were asked to participate. However, there may very well be differences regarding time allocated to preventive activities and tobacco preventive work by the PDS in the different counties in Norway. Because such differences cannot be excluded, the results may not be generalizable to the whole country. The response rate of 53.4% may have introduced some selection bias. Nonresponding dental professionals may be those who perceive the most time pressure and thus did not take the time to participate. Accordingly, the same nonresponders are perhaps more likely being inactive for the same reason regarding preventive snus use intervention. In both cases, our study may overestimate intervention activity in the PDS.

In overall, the present study shows two major findings: the minimal intervention method for tobacco prevention and cessation is little known among dental professionals in south‐eastern Norway, and the PDS is an underutilized arena for prevention of snus use. Increased knowledge about brief intervention methods and the possibility to allocate time to tobacco preventive work is essential to achieve changes.

## CONFLICT OF INTEREST

The authors have no conflict of interest to disclose.
